# Spatial–temporal clustering of malaria using routinely collected health facility data on the Kenyan Coast

**DOI:** 10.1186/s12936-021-03758-3

**Published:** 2021-05-20

**Authors:** Alice Kamau, Grace Mtanje, Christine Mataza, Philip Bejon, Robert W. Snow

**Affiliations:** 1 KEMRI-Wellcome Trust Research Programme, Nairobi, Kenya; 2 Ministry of Health, Kilifi County Government, Kilifi, Kenya; 3 Centre for Tropical Medicine and Global Health, Nuffield Department of Clinical Medicine, University of Oxford, Oxford, UK

**Keywords:** Malaria, Hotspots, Spatial clusters, Spatial–temporal dynamics, Heterogeneity

## Abstract

**Background:**

The over-distributed pattern of malaria transmission has led to attempts to define malaria “hotspots” that could be targeted for purposes of malaria control in Africa. However, few studies have investigated the use of routine health facility data in the more stable, endemic areas of Africa as a low-cost strategy to identify hotspots. Here the objective was to explore the spatial and temporal dynamics of fever positive rapid diagnostic test (RDT) malaria cases routinely collected along the Kenyan Coast.

**Methods:**

Data on fever positive RDT cases between March 2018 and February 2019 were obtained from patients presenting to six out-patients health-facilities in a rural area of Kilifi County on the Kenyan Coast. To quantify spatial clustering, homestead level geocoded addresses were used as well as aggregated homesteads level data at enumeration zone. Data were sub-divided into quarterly intervals. Kulldorff’s spatial scan statistics using Bernoulli probability model was used to detect hotspots of fever positive RDTs across all ages, where cases were febrile individuals with a positive test and controls were individuals with a negative test.

**Results:**

Across 12 months of surveillance, there were nine significant clusters that were identified using the spatial scan statistics among RDT positive fevers. These clusters included 52% of all fever positive RDT cases detected in 29% of the geocoded homesteads in the study area. When the resolution of the data was aggregated at enumeration zone (village) level the hotspots identified were located in the same areas. Only two of the nine hotspots were temporally stable accounting for 2.7% of the homesteads and included 10.8% of all fever positive RDT cases detected.

**Conclusion:**

Taking together the temporal instability of spatial hotspots and the relatively modest fraction of the malaria cases that they account for; it would seem inadvisable to re-design the sub-county control strategies around targeting hotspots.

**Supplementary Information:**

The online version contains supplementary material available at 10.1186/s12936-021-03758-3.

## Background

The Pareto principle states that for many outcomes approximately 80% of consequences come from only 20% of the causes. The concept was developed under economic theory but has been applied to models of infectious disease epidemiology [1–3]. In malaria, events are often over-distributed in space and time driven by variations in local vector ecology, host susceptibility to infections or outcomes of infections [1, 4–12].

The concept that malaria is over-distributed in space has led to attempts to define “hotspots” in more stable, endemic areas of Africa [13]. The active detection of cases or infection linked to household coordinates of population censuses provides valuable insights on the extent and frequency of definable hotspots of potential disproportionately high burden/clustered households. Under stable endemic settings that remain under the control phases, passive case detection (PCD) data from routine health information systems is all that is typically available to define households or local areas with the highest burdens. However, the use of routine health facility data for ‘hotspots’ detection remains underutilized in these areas due to their limitations in terms of representativeness and completeness [14]. Hence, far fewer studies have investigated the potential of passive case detection in health facilities to identify hotspots [10, 15–21].

As we move towards improving the use of routine data, the use of spatial tools to detect malaria hotspots (i.e. single villages or groups of households within villages with increased risk of malaria transmission) using routinely collected data would increase the value of local health information systems at district levels. Previous studies have tended to use geospatial data at the household level, which is not available in routine reporting, where location data is usually restricted to village or enumeration zone. Here the objective was to explore the spatial and temporal dynamics of fever positive rapid diagnostic test (RDT) malaria cases routinely collected in six health facilities along the Kenyan Coast.

## Methods

### Study area

**Fig. 1:**
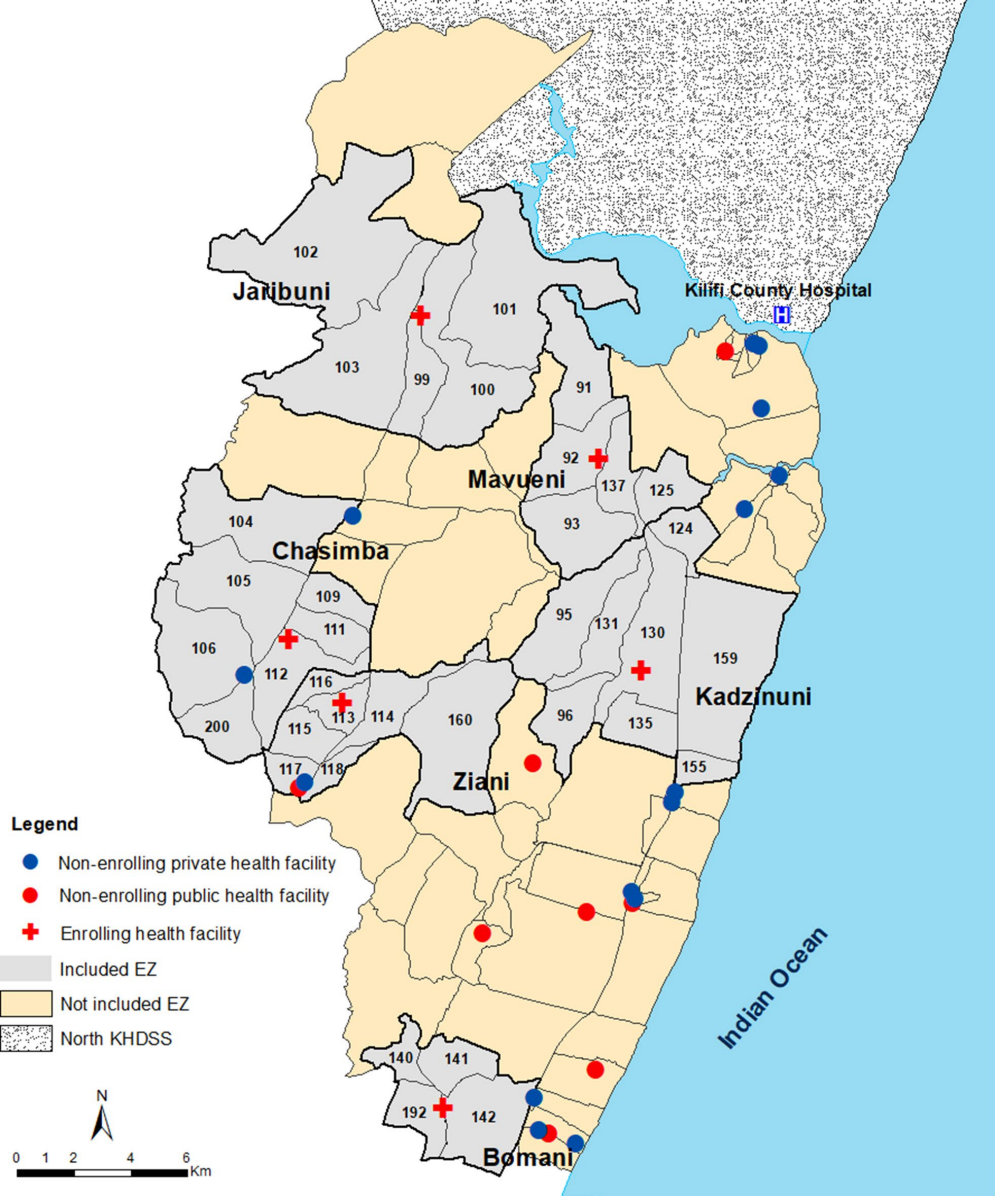
Map showing the location of the health facilities and their catchment areas

This is a secondary analysis of data collected from six health facilities located in the southern part of Kilifi Health and Demographic Surveillance System (KHDSS) located along the Kenyan coast (Fig. 1). The study area has been described in detail elsewhere [22, 23]. Briefly, malaria transmission is perennial but relatively higher during the long (April-June) and short (October-December) rains with an infection prevalence of approximately 10% detectable among residents of all ages [22]. In this area, 29 peripheral private and public health facilities and one referral hospital (Kilifi county hospital) provide health care to the population. The six health facilities were selected on the basis that they were public health facilities and were more likely to comply with government policies on diagnosis, treatment and participate in routine reporting of data. They also had a high burden of patients (a minimum of 10 patients per day) and were not part of ongoing active surveillance. A catchment area for the health facilities was defined as the enumeration zones (EZs) within a 2 km radius of each health facility. The catchment areas were estimated as the boundary within which the probability of attending these health facilities was relatively high. The area included an enumerated mid-year population of 72,560 in 2018 and 36 EZs consisting of 9,596 homesteads.

### Health facility‑based passive surveillance of fever infections

At each facility, the study included records of all patients ≥ 6 months of age that sought treatment between March 2018 to February 2019 with a history of fever in the last 24 h or a measured axillary temperature ≥ 37.5 °C, hereafter referred as febrile patients. All febrile patients were tested using a malaria rapid diagnostic test (RDT) (CareStart™) to detect HRP2 specific to *Plasmodium falciparum*. If the RDT results were positive the patient received appropriate treatment as per the Government of Kenya guidelines for malaria-case management [24]. During the surveillance period, malaria test positivity rate (TPR) did not differ during the wet (42.7%) versus dry season (43.5%) (p = 0.173) [22].

### Spatial resolution

To quantify spatial clustering of passively detected cases, two spatial resolutions were considered. All health facility attendees included in this study were linked to the KHDSS homestead level geospatial coordinates in order to assess heterogeneity at very fine spatial scale (i.e. the gold standard methodology). However, geospatial data at this level would not be available routinely in health facilities. Since patient records generally do not include actual residential addresses, homesteads level data was aggregated at enumeration zone (EZ) (equivalent to village) in order to explore the feasibility of hotspots detection using village level information. The geographic coordinates of the centroid of each EZ was used.

### Statistical analysis

The focus of the analysis was across all ages (≥ 6 months) as these data are available from existing DHIS2 platforms and because TPR across all ages was strongly associated with infection prevalence in the community, suggesting that passive surveillance does provide a reflection of infection prevalence in the community [22]. Since geospatial coordinates (longitude and latitude) of homesteads tended to have small number of patients resulting in higher standard errors and therefore less precise TPR, smoothing was performed for visualization purposes only on the maps. TPR was calculated as a simple proportion of RDT positive homestead members within a radius of < 1 km around each index patient. To explore different smoothed estimates of TPR, the radius was also altered at < 0.2 km and < 0.5 km. TPR for each EZ was calculated as the number of positive diagnostic tests as a proportion of the total tests performed among febrile patients aggregated at the EZ level.

### Local spatial cluster detection

Local clustering detection was performed using Martin Kulldorff’s spatial scan statistic (SaTScan) [25]. The raw data, and not smoothed TPRs, were used for hotspot detection. SaTScan imposes circular scanning windows across a study area with radius varying from zero to a maximum of 50% of the population in the sampling frame. An elliptic window shape can be used as an alternative to the circular window, in which case a set of ellipses with different shapes and angles are used as the scanning window. This may provide higher power for true clusters that are elliptical in shape, but lower power for circular or other very compact clusters [25]. Each scanning window is evaluated as a potential cluster by the calculation of a log likelihood ratio (LLR) test statistic based on the observed, expected and total number of cases. To test the null hypothesis of complete spatial randomness, SaTScan employs Monte Carlo simulations where for each simulation run, the observed cases are randomly permuted in space across the entire set of data locations. The observed log likelihood is then compared with the simulated log likelihoods to determine significance. The statistical significance of a cluster (or ‘‘hotspot’’) is then evaluated taking into account the multiple tests for the many potential cluster locations and sizes assessed.

Kulldorff’s spatial scan statistics using Bernoulli probability model was used to detect hotspots of fever positive RDTs, where cases were febrile individuals with a positive test and controls were individuals with a negative test. The maximum spatial cluster size was set at a radius of 3 km. For each detected hotspot, a relative risk (RR) was computed. The RR is the magnitude of the risk of malaria for individuals residing within the hotspot compared to the estimated risk in the surrounding area. The circular windows were used in line with common practice. They are computationally efficient, and it is easier to determine hotspot properties that are of interest (Radius, RR and significance). The most likely cluster (hereafter referred to primary cluster) was identified based on the maximum log likelihood ratio. In addition, other clusters with statistically significant log likelihood values were defined as secondary clusters.

### Temporal stability of hotspots

There were no clear patterns between seasonality and TPR [25]. Therefore, to test for temporal stability of spatial clusters, the data was sub-divided into quarterly intervals i.e. March to May 2018 (Q1), June to August 2018 (Q2), September to November 2018 (Q3) and December 2018 to February 2019 (Q4). The spatial analysis was repeated for each interval rather than a spatial–temporal because the size of the database made secondary clusters very likely and the option for analysing secondary clusters is not available for spatial–temporal analysis but is validated for spatial-only analysis [26].

Spatial cluster analysis was performed using SaTScan™ software version 9.6 (Information Management Services Inc, Silver Spring, Maryland, USA). All other statistical analyses were performed in Stata, version 13 (Stata Corporation, College Station, TX) and R version 3.6.1 (R Core Team (2019), Vienna, Austria). Maps of hotspots were produced in R.

## Results

### Description of the spatial data

Overall, the study comprised of 28,134 febrile health facility attendees across all ages in 5,323 geocoded homesteads between March 2018 and February 2019. Among all febrile patients, 12,143 (43%) tested positive for malaria using RDT. The number of febrile health facilities attendees varied across the quarterly intervals ranging between 4,284 and 9,119 (Table 1). The RDT fever test positivity rate was lowest between March and May 2018 at 39% (1,651 cases) and highest between June and August 2018 at 45% (4,096 cases) (Table 1). A similar test positivity rate (45%) was observed between December 2018 and February 2019 (Table 1). The overall smoothed 1 km TPR ranged between 0% and 89.3% in 5,323 geocoded homesteads located in 36 EZs (Additional file 1). The smoothed TPR was comparable when the radial distance was altered which ranged between 0 and 100% for both 0.2 km and 0.5 km (Additional file 1). The 1 km radius was chosen over other radial distances as ‘noise’ was minimized with greater stability.

**Table 1:** Summary of data routinely collected at six health facilities stratified by quarterly intervals

Summary measures	March–may 2018 (Q1)	June–august 2018 (Q2)	September–november 2018 (Q3)	December 2018–february 2019 (Q4)	Overall
Number of geo-coded homesteads	2290	3532	3368	2890	5323
Number of attendees (N)	4284	9119	8396	6335	28,134
Number of cases (n)	1651	4096	3554	2842	12,143
TPR, (95% CI)	38.5% (37.1%, 40.0%)	44.9% (43.9%, 45.9%)	42.3% (41.3%, 43.4%)	44.9% (43.6%, 46.1%)	43.2% (42.6%, 43.7%)

### Spatial hotspot detection

**Fig. 2:**
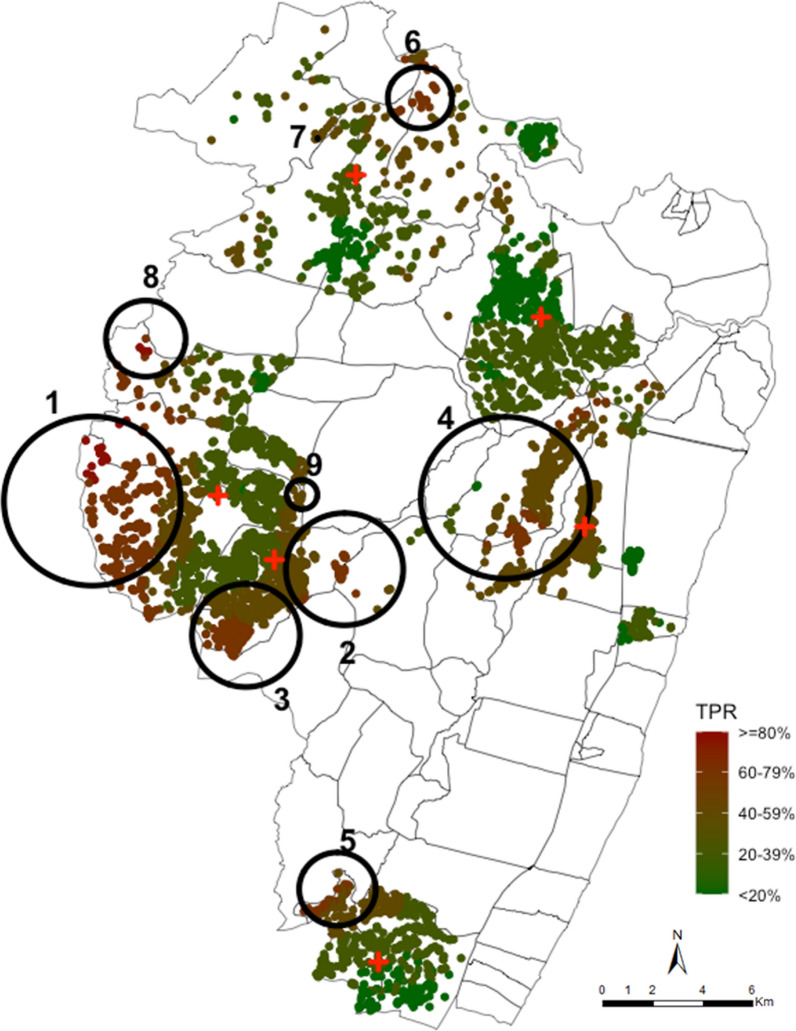
Spatial distribution of smoothed mean TPR across 12 months of surveillance at homesteads level aggregated at 1 km radius and the spatial hotspots of fever positive RDT cases, analysed without smoothing. Each plotted point represents an individual homestead, where red shading indicates high TPR and green shading indicates lower TPR. The large black circles indicate the significant hotspots (analysed without smoothing) where 1 indicates the primary cluster located in Chasimba health centre and clusters 2–9 are the secondary hotspots located in Ziani, Kadzinuni, Bomani and Jaribuni dispensaries

**Fig. 3:**
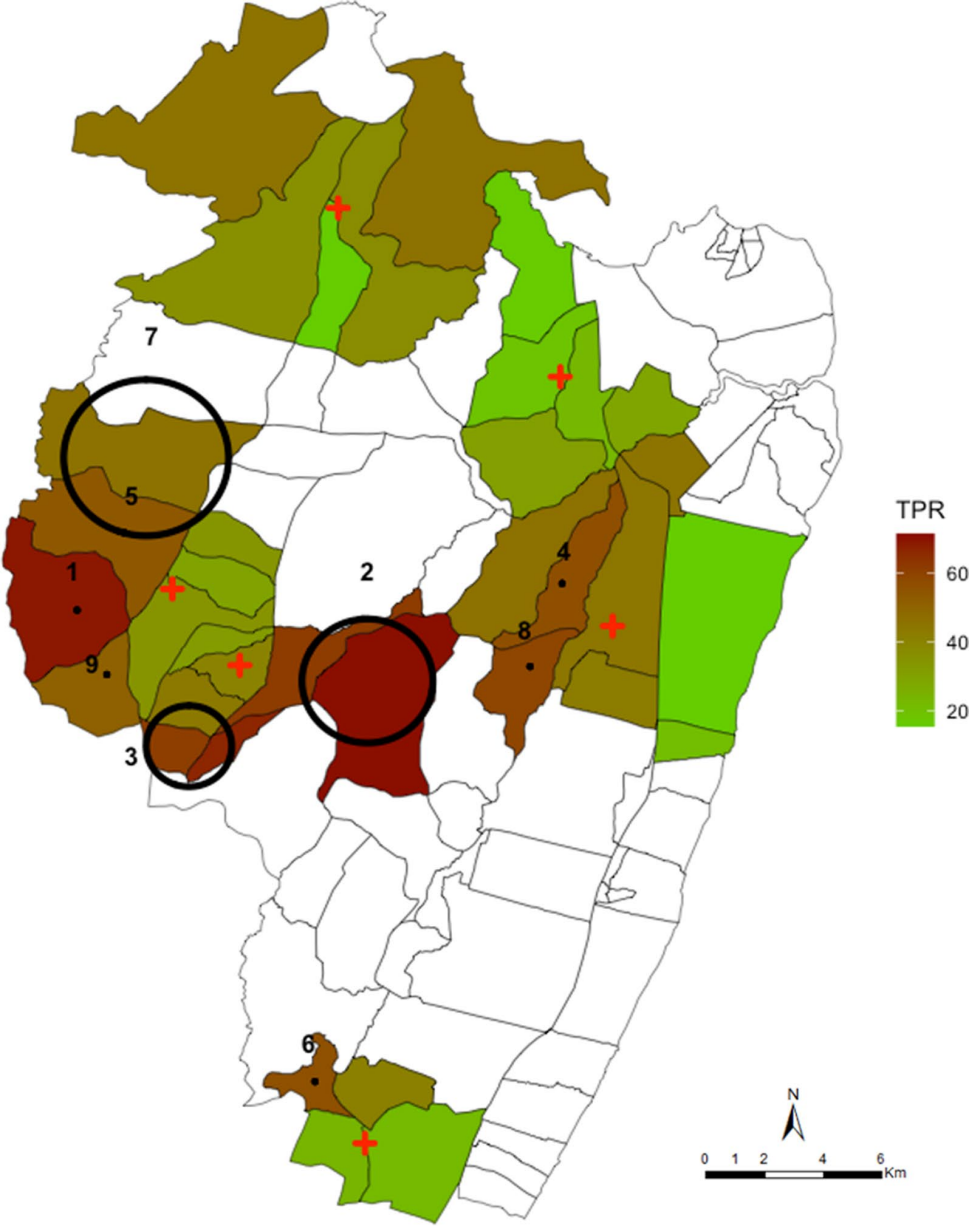
Spatial pattern of annual malaria RDT positivity rate by enumeration zones and spatial hotspots of fever test positive cases. The red shading in the choropleth map indicates very high TPR (≥ 60%), brown indicated high TPR (40–59%), dark green is low-moderate TPR regions (20–39%) and light green shading indicating lower TPR (< 20%). The large black circles indicate the location of high-risk clusters detected by purely spatial scan statistics using geographic coordinates of the centroid of each EZ. Cluster 1 indicates the primary cluster located in Chasimba health centre and clusters 2—9 are the secondary hotspots located in Ziani (clusters 2 and 3), Kadzinuni (clusters 4 and 8) and Bomani (cluster 6) dispensaries. The additional secondary clusters 5, 7 and 9 were located in Chasimba health centre

Across 12 months of surveillance, there were nine significant clusters that were identified using the purely spatial scan statistics among RDT positive fevers of all age groups (Fig. 2). The primary cluster with a radius of 3.0 km was detected in the west of the study area among patients attending Chasimba health centre, composed of 289 (5.4%) homesteads accounting for 16.0% (1939) of all fever test positive cases detected (cluster 1 in Fig. 2). The homesteads within this cluster were 1.75 (p < 0.001) times more at risk of testing positive for malaria than homesteads outside the cluster. In addition, secondary hotspots were identified among patients attending three of the five remaining health facilities (i.e. Ziani (cluster 2, 3, and 9), Kadzinuni (cluster 4), Bomani (cluster 5) and Jaribuni (cluster 6 and 7) dispensaries, Fig. 2). There were no clusters detected using data obtained from Mavueni dispensary. Cumulatively, in both the primary and secondary hotspots, there were 1520/5323 (28.6%) homesteads that accounted for 51.8% (6293/12,143) of all fever test positive cases detected in the study area. When data were aggregated at EZ level, a spatial resolution equivalent to what might be available through routine health information systems, the primary and secondary hotspots identified were generally located in the same areas as those identified using the homestead level data (Fig. 3).

### Temporal stability of hotspot detection

**Fig. 4:**
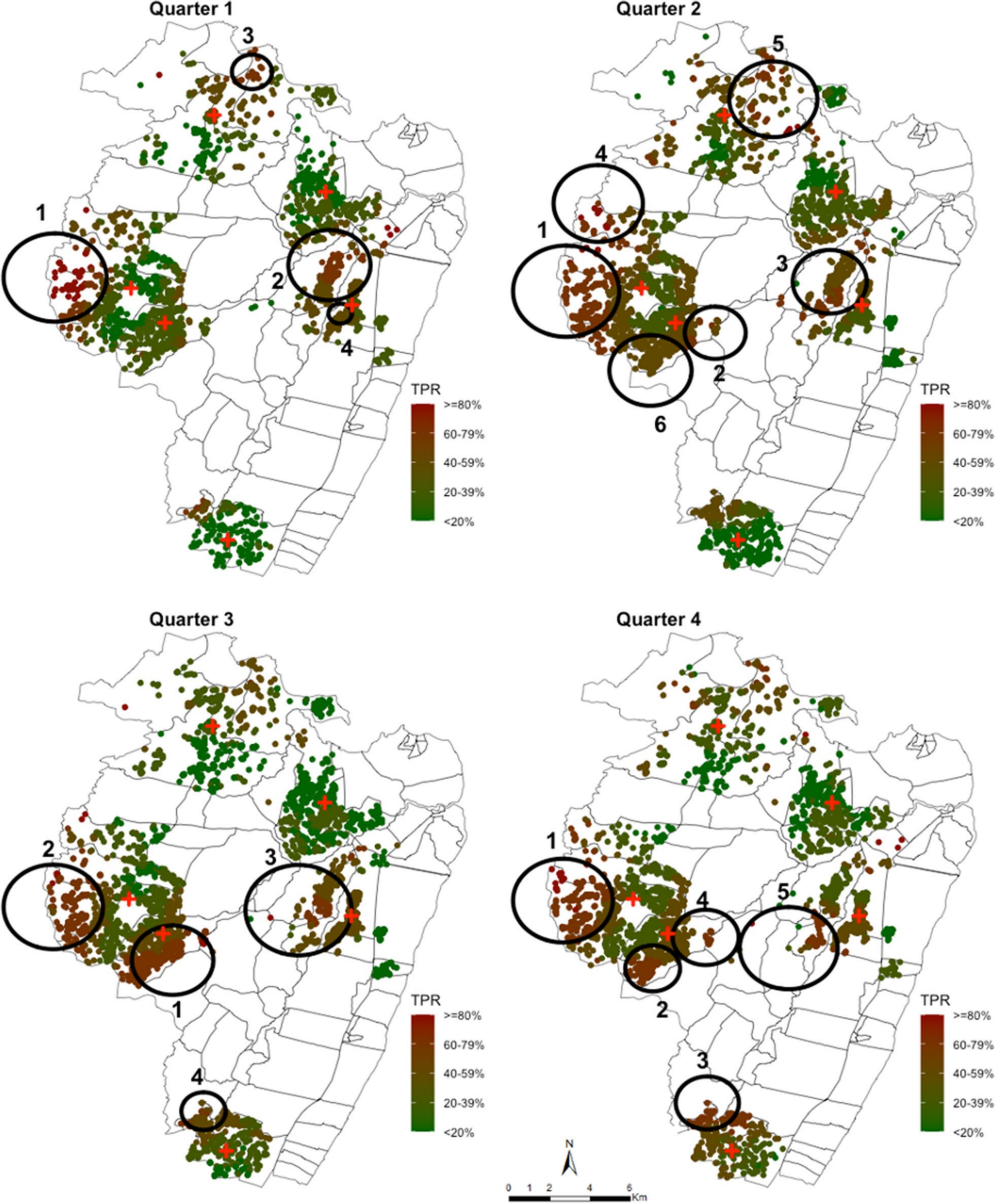
Spatial distribution of smoothed mean TPR across all ages at homesteads level aggregated at 1 km radius and the spatial hotspots of fever positive RDT cases stratified into quarterly monthly periods, analysed without smoothing. Each plotted point represents an individual homestead, where red shading indicating high TPR and green shading indicating lower TPR. The large black circles indicate the significant hotspots (analysed without smoothing) where 1 indicates the primary cluster and clusters 2–6 are the secondary hotspots

To test for temporal stability of spatial clusters, the data was sub-divided into quarterly intervals. When the spatial analysis was repeated for each quarter, several hotspots were detected. The relative risk of infection within these clusters was significantly higher in comparison to the population outside of these clusters (Table 2). The radius of the spanning windows ranged from 0.66 km to 3.0 km and four, six, four and five clusters were detected in the first, second, third and four quarters, respectively (Table 2). These spatial clusters represented between 17.7% (405) and 32.6% (1,098) of all the geocoded homesteads (Table 2). Only two clusters seemed temporally stable across the quarterly intervals (Fig. 4).

**Table 2:** Spatial clusters of fever positive RDT cases detected by SaTScan, ordered from the cluster with the highest LLR, stratified by quarterly intervals

Period	Cluster order	Radius (km)	Number of cases in cluster (n)	Cumulative cases/total cases	Expected cases	Number of HMs in cluster (n)	Cumulative HMs/total HMs	RR	p-value
Q1	1	2.90	287	287/1651	139.12	106	106/2290	2.29	< 0.001
	2	2.31	270	557/1651	174.58	216	322/2290	1.65	< 0.001
	3	1.13	44	601/1651	21.2	23	345/2290	2.11	< 0.001
	4	0.66	88	689/1651	59.74	60	405/2290	1.50	0.03
Q2	1	3.00	583	583/4096	371.51	220	220/3532	1.66	< 0.001
	2	1.72	289	872/4096	197.21	143	363/3532	1.50	< 0.001
	3	2.09	363	1235/4096	270.88	227	590/3532	1.37	< 0.001
	4	2.52	103	1338/4096	64.69	40	630/3532	1.61	< 0.001
	5	2.49	222	1560/4096	163.97	84	714/3532	1.37	< 0.001
	6	2.32	283	1843/4096	229.1	251	965/3532	1.25	0.02
Q3	1	2.28	815	815/3554	550.29	546	546/3368	1.62	< 0.001
	2	2.78	503	1318/3554	306.47	177	723/3368	1.75	< 0.001
	3	2.97	537	1855/3554	400.44	306	1029/3368	1.40	< 0.001
	4	1.24	133	1988/3554	89.74	69	1,098/3368	1.50	< 0.001
Q4	1	2.75	538	538/2842	334.22	216	216/2890	1.75	< 0.001
	2	1.51	226	764/2842	157.47	188	404/2890	1.47	< 0.001
	3	1.74	134	898/2842	84.34	74	478/2890	1.62	< 0.001
	4	1.82	247	1145/2842	183.04	178	656/2890	1.38	< 0.001
	5	2.70	114	1259/2842	73.12	76	732/2890	1.58	< 0.001

The primary cluster in the first quarter was 2.9 km radius west of the study area, composed of 106 (4.6%) homesteads accounting for 17.3% (287) of all fever test positive cases identified in that period. The homesteads within this cluster attended Chasimba health centre and were 2.29 (p < 0.001) times more at risk of testing positive for malaria than homesteads outside the cluster. In the second quarter, the primary cluster had the highest-risk (1.66; p < 0.001) and overlapped with the cluster detected in the first quarter. This cluster represented 8.5% of the homesteads accounting for 14.2% of all fever test positive cases (Table 2). The primary cluster detected in the third quarter had a radius of 2.28 km with a risk of 1.62 (p < 0.001), but was located in the central part of the study area among patients attending Ziani dispensary. This cluster represented 15.9% of the homesteads and 22.9% of all fever test positive cases detected in that period (Table 2). The cluster with the highest risk (RR = 1.75; p < 0.001) in the third quarter was in a similar location as the primary clusters detected in the first and second quarter. The fourth quarter showed a similar pattern to what was observed in the first and second quarter. The highest-risk hotspot was detected in the same location with a RR of 1.75 (p < 0.001) and represented 7.4% of the homesteads and accounted for 18.9% of all fever test positive cases in that period (Fig. 4).

There were several secondary hotspots identified across all the quarterly intervals (Table 2 and Fig. 4). The secondary clusters were characterized in Jaribuni, Kadzinuni and Bomani dispensaries. However, the spatial location and size varied across the intervals. There were neither primary nor secondary clusters detected using data obtained from Mavueni dispensary. Cumulatively, all the hotspots detected in the first quarter represented 17.7% (405/2290) of all the homesteads accounting for 41.7% (689/1651) of all fever test positive cases detected. In the second quarter 27.3% (965/3532) of the homesteads accounted for 45.0% (1843/4096) of the case, in the third quarter 32.6% (1098/3368) of the homesteads accounted for 56.0% (1988/3554) of the cases and in the fourth quarter 25.3% (732/2890) of the homesteads accounted for 44.3% (1259/2842) of the cases detected (Table 2).

**Fig. 5:**
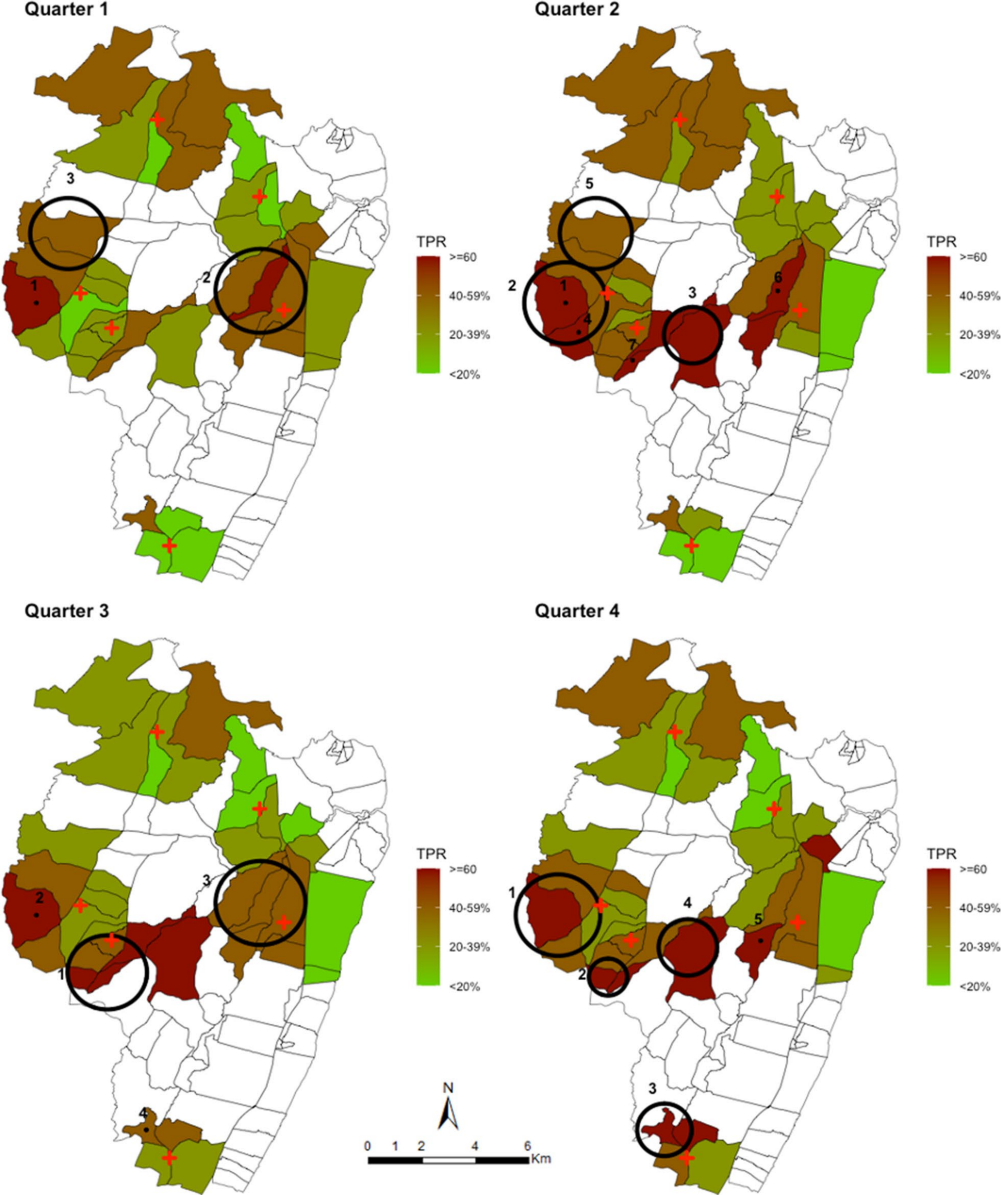
Spatial pattern of annual malaria RDT positivity rate by enumeration zones and spatial hotspots of fever test positive cases stratified into quarterly monthly periods. The red shading in the choropleth map indicates very high TPR (≥ 60%), brown indicated high TPR (40–59%), dark green is low-moderate TPR regions (20–39%) and light green shading indicating lower TPR (< 20%). The large black circles indicate the location of high-risk clusters detected by purely spatial scan statistics using geographic coordinates of the centroid of each EZ. Cluster 1 indicates the primary cluster located and clusters 2–7 are the secondary hotspots

In the two temporally stable hotspots, 142/5323 (2.7%) homesteads were consistently identified in these hotspots across the four-time intervals accounting for 10.8% (1311/12,143) of all fever test positive cases detected, 353 (6.6%) were identified three times and accounted for 17.5% (2122) of the cases, 218 (4.1%) identified twice accounting for 5.3% (643) of the cases and 306 (5.8%) were identified once accounting for 9.0% (1095) of the cases while 4,304 (80.9%) homesteads were in the unstable hotspots or were never identified in a hotspot area. Again, when data was aggregated at EZ (village) level, the primary and secondary hotspots identified across the quarterly intervals (Fig. 5) were roughly located in the same areas as those identified using the homestead level data (Fig. 4).

## Discussion

The results presented demonstrate that information obtained through routine testing of febrile patients for malaria can identify spatial and temporal heterogeneities of malaria risk at very fine spatial scales, where homestead coordinates are available and importantly at lower resolutions where village names might be available (Fig. 2, 3).

Fever positive RDTs cases exhibited spatial heterogeneity as evidenced by the existence of statistically significant (p < 0.05) spatial clusters in groups of homesteads among residence of six catchment areas of six health facilities. Across the 12-month surveillance period, these clusters included 52% of all fever positive RDT cases detected in 29% of the geocoded homesteads in the study area. In addition, the power to detect clusters of fever positive RDT cases did not diminish when data were spatially aggregated at EZ (village) level (Fig. 3) as demonstrated previously [27]. However, aggregations negatively impacted the ability to more accurately determine the exact spatial location of the clusters.

These results support the hypothesis that malaria tends to significantly cluster within certain geographic units [1, 5–7, 10, 11, 20, 21, 28].

When the temporal stability of the hotspots was examined, the hotspots varied greatly across the intervals (Fig. 4). The clusters of fever positive RDT cases moved in space across the quarterly intervals and often did not recur in the same location. This has also been noted in other clustering studies [10, 20, 21]. Only two temporally stable hotspots were identified with 2.7% of the homesteads consistently located in these areas across all the intervals and included 10.8% of all fever positive RDT cases detected.

Most studies that have mapped the spatial distribution of malaria burden have relied upon surveys of well-defined demographic and spatial distributions of the at-risk population [6, 10, 17, 20, 21, 26, 29, 30]. Few studies have examined the use of routine PCD data in defining clusters of disease risks [10, 15–21, 31, 32]. Of these PCD studies, variable patterns of spatial clustering have been reported. Some studies support consistent hotspots [19, 33] while others suggest greater variability [10, 17, 20, 21]. For example, in a highland area in Kenya the risk of malaria was consistently higher between 2001 and 2004 among individuals living in a hotspot area however, the number of households within the cluster varied and included between 29.3% and 49.3% of all cases detected [33]. In Ouagadougou, Burkina Faso the location of clusters identified as high risk varied little across three transmission periods [19]. In areas of moderate malaria transmission in coastal Kenya [10, 17], very low transmission in a highland area of Kenya [21] and Nanoro DSS, Burkina Faso [20], hotspots of malaria were not consistently identified over time. At much lower transmission health facility sites, authors were unable to identify any statistically significant clustering of cases [31, 32], although this may reflect diminished power due to the lower number of cases at lower transmission, as meta-analysis suggests that the effect size of clustering of cases becomes more marked at lower transmission [13].

The use of passively collected routine health facility data does offer opportunities to detect clusters down to the village level at an affordable cost. However, appropriately structured and defined health facility data will be needed. In many areas, routinely collected health facility data generally do not include accurate residential address. Therefore, to tap the full potential of these data, it will be important to refine the current surveillance tools such that they have the potential of collecting information at sufficiently precise scales (at individual level and ‘village’ scales). Closing these gaps could result in health information systems that have the potential of becoming scalable, integral, and sustainable components of control programmes, which can then target interventions to clusters of elevated risk [18, 21].

Hotspot-targeted interventions have been hypothesized to be a highly efficient method of reducing malaria transmission not only inside these hotspots but also in adjacent areas [7, 10]. While biologically plausible, there has been mixed evidence to support this concept. For example, a trial conducted in western Kenya failed to observe any sustained reduction in transmission [34]. Despite achieving high coverage of interventions in hotspot areas, the interventions resulted in a modest and transient reduction in transmission inside targeted hotspots and failed to influence malaria transmission dynamics outside the targeted areas [34]. In a more recent study in Rufiji District, southern Tanzania, the implementation of a locally tailored surveillance-response strategy contributed convincingly to the reduction of malaria burden in hotspot villages (with the highest malaria incidence ratio) using health facility-based data [35]. This study offered the first example of surveillance as an intervention in areas with high malaria burden, which is in line with the current World Health Organization-recommended strategies for district-level malaria information systems [35–37].

## Limitations

The data presented here does not represent the universe of all the health facilities in the study area, febrile patients may have elected to use home-based treatment, private facilities or formal health services more distal to their home. The data do however represent information from busy public health facilities and were used here to demonstrate the potential value of information obtained through routine testing of febrile patients for malaria in describing the local malaria epidemiology at fine spatial scales. The spatial and temporal coverage of observations in this study is likely to have had an impact on the stability of the hotspots detected. However, PCD data needs analysis over short temporal resolutions to instigate immediate intervention requiring real-time analysis. In the present analysis, hotspots stability was based on the fraction of homesteads that fell within clusters that occurred across all the four-time intervals. Lack of longer-term data in this study limits the ability to examine stability of hotspots beyond a year. It is possible that the spatial heterogeneity observed may have been due to measurement bias of how fever positive RDTs cases were defined. For example, there is a possibility that a fever test positive case was from a single infectious bite or repeated inoculations within the same individual because all cases testing RDT positive including re-attendance were used. To evaluate the degree of potential measurement bias as an alternative explanation, SaTScan analysis was rerun using records of first cases only of RDT positive patients. The results were generally similar to the results obtained using records of all cases (Additional file 2). Although Kulldorff’s scan statistic was successfully used to detect circular clusters, it may ignore more subtle small-scale spatial clusters that do not fit within circular windows [38, 39]. Arguably, clusters that require specific window shapes are relatively subtle and therefore unlikely to be of primary importance to routine malaria control. Many of the limitations of this study would apply to routine health facility data in many settings, and this study was set out to test the utility of such data for hotspot detection.

## Conclusion and programme implications

In this study, approximately a third of the homesteads in the study area fell within identified hotspots and accounted for half of all health facility fever positive RDTs cases. These hotspots varied over time, with only two temporally stable hotspots which accounted for 10.8% of malaria cases in the 2.7% of homesteads.

The operational question is whether these areas are detectable with routine data and, if so, whether they are ‘hot enough’ to re-design district level control strategies from one of universal coverage of interventions, assuming no heterogeneity, to one of a more tailored, nuanced approach based on local data. The temporal instability of the hotspots suggests that the use of local data would require real-time analysis and intervention. The complexity of these analyses in time and space suggests that hotspot-targeted interventions may at this stage be unnecessary in this part of Kilifi county.

The results presented continue to demonstrate that information obtained through routine testing of febrile patients for malaria can describe local malaria epidemiology at fine spatial scales. The challenge remains to develop programmatically affordable and scalable approaches using routine data that allows for the identification of local spatial heterogeneity to consider targeted supplementary control efforts.

## Supplementary Information

**Additional file 1:** Panel A shows the distribution of yearly smoothed mean TPR aggregated at a 1 km radius for all ages. Panel b shows the distribution of yearly smoothed mean TPR aggregated at a 0.5 km radius for all ages. Panel c shows the distribution of yearly smoothed mean TPR aggregated at a 0.2 km radius for all ages.

**Additional file 2:** Spatial distribution of smoothed mean TPR across all ages using records of first cases only of RDT positive patients at homesteads level aggregated at 1 km radius, the spatial hotspots of fever test positive cases and the location of the health facilities.

## Data Availability

Data cannot be shared publicly because it includes homestead level coordinates as an essential component, and these are personal identifiable data. Data that support the findings of this study are available from the KEMRI Institutional Data Access/Ethics Committee. Details of the guideline can be found in the KEMRI-Wellcome data sharing guidelines (https://kemri-wellcome.org/about-us/#ChildVerticalTab_15). Access to data is provided via the KEMRI Wellcome Data Governance Committee: dgc@kemri-wellcome.org.

## Abbreviations

RDT: Rapid diagnostic test
KHDSS: Kilifi health and demographic surveillance system
EZ: Enumeration zones
TPR: Test positivity rate
SaTScan: Martin Kulldorff’s spatial scan statistic
LLR: Log likelihood ratio
RR: relative risk
PCD: Passive case detection
WHO: World Health Organization
HRP2: Histidine-rich protein 2
DHIS2: District health information system version 2
DSS: Demographic surveillance syste
