# Supplementary material for: Spatial–temporal clustering of malaria using routinely collected health facility data on the Kenyan Coast
Source: Malar J. 2021 May 20;20:227. doi: 10.1186/s12936-021-03758-3 (PMC8138976; doi:10.1186/s12936-021-03758-3)
Supplement: Supplementary file 1 — Additional file 1: Panel A shows the distribution of yearly smoothed mean TPR aggregated at a 1 km radius for all ages. Panel b shows the distribution of yearly smoothed mean TPR aggregated at a 0.5 km radius for all ages. Panel c shows the distribution of yearly smoothed mean TPR aggregated at a 0.2 km radius for all ages. [file 12936_2021_3758_MOESM1_ESM.docx]

**Additional Files**

**Additional file 1:** Panel A shows the distribution of yearly smoothed mean TPR aggregated at a 1 km radius for all ages. Panel b shows the distribution of yearly smoothed mean TPR aggregated at a 0.5 km radius for all ages. Panel c shows the distribution of yearly smoothed mean TPR aggregated at a 0.2 km radius for all ages
