# Supplementary material for: Spatial–temporal clustering of malaria using routinely collected health facility data on the Kenyan Coast
Source: Malar J. 2021 May 20;20:227. doi: 10.1186/s12936-021-03758-3 (PMC8138976; doi:10.1186/s12936-021-03758-3)
Supplement: Supplementary file 2 — Additional file 2: Spatial distribution of smoothed mean TPR across all ages using records of first cases only of RDT positive patients at homesteads level aggregated at 1 km radius, the spatial hotspots of fever test positive cases and the location of the health facilities. [file 12936_2021_3758_MOESM2_ESM.docx]

**Additional file 2**: Spatial distribution of smoothed mean TPR across all ages using records of first cases only of RDT positive patients at homesteads level aggregated at 1 km radius, the spatial hotspots of fever test positive cases and the location of the health facilities.


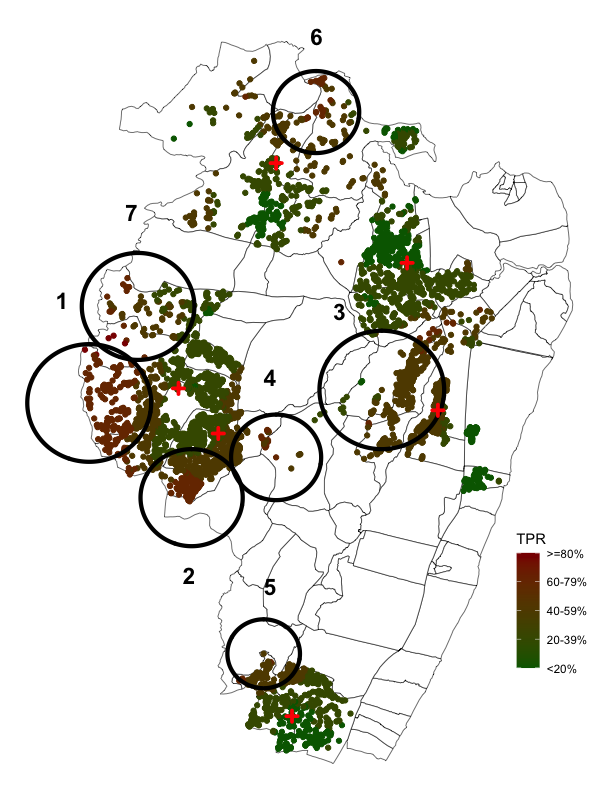


Each plotted point represents an individual homestead, where red shading indicating high TPR and green shading indicating lower TPR. The large black circles indicate the significant hotspots (analysed without smoothing) where 1 indicates the primary cluster located in Chasimba health centre and clusters 2 - 9 are the secondary hotspots located in Ziani, Kadzinuni, Bomani and Jaribuni dispensaries.
